# Research History, Pathology and Epidemiology of Ossification of the Posterior Longitudinal Ligament and Ligamentum Flavum

**DOI:** 10.3390/jcm11185386

**Published:** 2022-09-14

**Authors:** Kenichiro Sakai, Toshitaka Yoshii, Takeo Furuya, Masaaki Machino

**Affiliations:** 1 Department of Orthopedic Surgery, Saiseikai Kawaguchi General Hospital, 5-11-5 Nishikawaguchi, Kawaguchishi 332-8558, Japan; 2 Department of Orthopedic Surgery, Tokyo Medical and Dental University, 1-5-45 Yushima, Tokyo 113-8519, Japan; 3 Department of Orthopedic Surgery, Chiba University Graduate School of Medicine, 1-8-1 Inohana, Chiba 260-8670, Japan; 4 Department of Orthopedic Surgery, Nagoya University Graduate School of Medicine, 65 Tsurumaicho, Nagoya 466-8550, Japan

## 1. Research History

Ossification of the spinal ligaments was first reported by Key in 1838 [1], based on an autopsy that revealed cervical spinal cord compression due to ossification of the posterior longitudinal ligament (OPLL) of the cervical spine. However, this disease only began to receive attention about 100 years later, in 1942, when Oppenheimer reported 18 cases of calcification or ossification of the anterior and posterior longitudinal ligaments [2]. Even at this time, the clinical significance of ossification of the spinal ligament was not yet widely recognized. The ossification of the spinal ligaments later became widely known in Tsukimoto’s report of cervical OPLL in 1960 [3]. The autopsy revealed ectopic bone formation adjacent to the posterior vertebral body, which was presumed to have originated from the posterior longitudinal ligament. In addition, Tsukimoto speculated that repeated minor trauma to the cervical spine induced the ectopic bone formation and noted the involvement of vascular factors. This report led to widespread recognition of ossification of the spinal ligaments and many studies on this condition.

In Japan, ossification of the spinal ligaments was listed in 1975 as a government-designated intractable disease, and the guideline for the treatment of cervical OPLL were first published in 2005. In 2019, the guideline was published for the treatment of ossification of spinal ligaments, which includes thoracic OPLL and ossification of the ligamentum flavum (OLF) [4].

## 2. Pathology

The mechanism of ossification of the spinal ligament occurs by two processes. First, endochondral ossification begins with chondrocyte proliferation in the deep layers of the posterior longitudinal ligament at the vertebral body attachment, resulting in ossification with intravascular invasion of chondrocytes. Second, connective tissue ossification occurs, in which fibroblast proliferation, hyperplasia, and vascular invasion develops in the entire area of the degenerated ligament, resulting in ossification [5].

The association of genetic predisposition with OPLL is evident from previous studies of household surveys, twin studies, human leukocyte antigen haplotyping, and analysis of etiologic genes. Several factors have been reported, including but not limited to the following: fibroblast growth factor 2 and fibroblast growth factor receptor 1; bone morphogenetic proteins 2 and 9; transforming growth factor β1 and β3; collagen types XVII α1 chain, VI α1 chain, and XI α2 chain; interleukin 1β and interleukin 15 receptor subunit α; Toll-like receptor 5; runt-related transcription factor 2; estrogen receptors 1 and 2; human leukocyte antigen haplotype; and vitamin D receptor [5]. In addition, six disease susceptibility genes were reported in the genome-wide association study in Japan [5]. One of these genes is the site encoding the *R-spondin 2* gene, which suppresses early chondrogenic differentiation by activating Wnt-β-catenin signaling. Genetic studies using single-nucleotide polymorphism analysis of OLF have reported an association with collagen type VI α1 chain [6].

In addition to genetic background, both diet and comorbidities may be associated with the development of and increased ossification of the spinal ligaments, as investigated in many studies. Although direct causal relationships have not yet been determined, OPLL may be associated with foods such as legumes, excessive vitamin A intake, disorders of calcium or glucose metabolism, and myotonic dystrophy [5]. Mechanical stress on the cervical spine may also be a relevant factor. Indeed, mechanical stress increases the expression of osteogenic differentiation genes in ligament cells of OPLL patients, and ossification progression is suppressed or reduced after cervical fusion surgery. Studies have reported an association of OLF with glucose metabolism disorder, myotonic dystrophy, and mechanical stress [6]. Furthermore, bone metabolism markers, sclerostin, fibroblast growth factor 23, fibronectin, leptin, pentosidine, and high-sensitivity C-reactive protein may be relevant biomarkers of spinal ligament ossification, but they have yet to be identified as definitive biomarkers [5].

## 3. Epidemiology

Many reports have described racial differences in the incidence of cervical OPLL, with a higher incidence in Asian individuals compared with other populations. Based on imaging studies, the incidence is 1.0–4.3% in East Asian and 0.1% in Caucasian individuals on simple radiographic studies, and 4.8% in Asian, 1.3% in Caucasian, 1.9% in Hispanic, and 2.1% in African individuals on computed tomography (CT) [7]. The incidence of thoracic OPLL is lower than that of cervical spine, reported to be 0.5–0.8% on simple radiographic studies and 1.6–1.9% on CT in Japanese individuals [8], whereas the incidence of thoracic OLF ranges from 4.8–6.2% on simple radiographic studies and 12–64% on CT [6].

Regarding sex differences, the incidence of cervical OPLL is approximately twice as high in men versus women; conversely, the incidence of thoracic OPLL is 1.5–3 times higher in women [7,8]. Thoracic OLF has been reported to be more common in men and vice versa, but there is no definitive recognition at present [6]. In addition, extensive ossification, such as spreading to the whole spine, is more common in women [7]. Regarding age of onset, cervical OPLL is more common after 50 years, thoracic OPLL after 40 years, and thoracic OLF beginning after 50–60 years [6,7,8].

Spinal ligament ossifications such as OPLL and OLF are closely related to each other and to other spinal ligaments, such as anterior longitudinal ligament ossification and supraspinous ligament ossification. On whole-spine CT in patients with cervical OPLL, 56.2% had thoracolumbar OPLL, more than 60% had thoracic OLF, 29% had supraspinous ligament ossification, and 55.8% had nuchal ligament ossification; moreover, the severity of cervical OPLL was associated with the ossification of these other ligaments [5]. In patients with thoracic OLF, 18% had cervical OPLL and 6% had thoracic OPLL [6].
